# Racial Differences in the Effect of Alzheimer’s Disease on Adherence to Medication Therapy for Chronic Diseases

**DOI:** 10.1093/geroni/igab046.231

**Published:** 2021-12-17

**Authors:** Arseniy Yashkin, Anatoliy Yashin, Galina Gorbunova, Igor Akushevich

**Affiliations:** 1 Duke University, Morrisville, North Carolina, United States; 2 Duke University, Durham, North Carolina, United States

## Abstract

Multiple dementia (the presence of one or more types of dementia in a single individual) and multi-morbidity (the presence of multiple chronic diseases in an individual) present a major challenge to the U.S. healthcare system. The reduction in cognitive function associated with neurocognitive disorders such as Alzheimer’s Disease (AD) and Related Dementias (ADRD) reduce the ability of the affected individual to take care of him/herself. This can manifest as reduced adherence to medication regimens designed to manage other chronic conditions, in reduced ability to engage in healthy behavior such as exercise, or in other ways. The result is an increase in the probability of otherwise avoidable adverse health outcomes and related healthcare costs. In this study, we showcase two high impact chronic conditions common in the elderly: hypertension and type 2 diabetes mellitus (T2D). Using a 5% sample of the total Medicare population we identify groups of individuals with AD/ADRD and i) hypertension, ii) T2D or iii) both. Each group is then propensity-score-matched to similar individuals with hypertension, T2D or both but without a diagnosis of AD/ADRD. The primary explanatory variable of interest is the medication possession ratio (MPR) calculated at 1-year intervals for prescribed diabetes and/or hypertension medications. MPRs were compared between the two groups using t-tests and standardized differences each year after baseline and until death/censoring. A Cox proportional hazard model was then used to estimate differences in survival between these two groups and across race/ethnicity-related strata. Reduced adherence with time and notable race/ethnicity-related differences were identified.

